# Variant Allelic Frequency to Track Therapy Response and Evaluate Leptomeningeal Disease in Metastatic Central Nervous System Cancers

**DOI:** 10.3390/diagnostics16060851

**Published:** 2026-03-13

**Authors:** Vindhya Udhane, Alexandra Larson, Jennifer N. Adams, Rakshitha Jagadish, Anthony Acevedo, Brett A. Domagala, Samantha A. Vo, Tarin Peltier, Daniel Sanchez, Viriya Keo, Julianna Ernst, Kala F. Schilter, Qian Nie, Honey V. Reddi

**Affiliations:** Belay Diagnostics, Suite 530, 1375 W. Fulton St, Chicago, IL 60607, USA

**Keywords:** leptomeningeal disease, variant allele frequency, longitudinal analysis, CSF liquid biopsy, Summit™

## Abstract

**Background**: Diagnosis of leptomeningeal disease (LMD) remains a clinical challenge due to nonspecific neurological symptoms, limitations of imaging, and the low sensitivity of cerebrospinal fluid (CSF) cytology. Molecular biomarkers, such as circulating tumor DNA (ctDNA) variant allele frequencies (VAFs), offer potential for improved detection and disease monitoring. **Methods**: Gene-level VAFs were analyzed from 118 Summit™ positive CSF specimens and evaluated in the context of clinical diagnosis, neurological presentation, neuroimaging, and CSF cytology. Longitudinal analyses were performed on serial CSF samples to assess VAF dynamics following therapy. **Results**: Longitudinal assessment demonstrated that decreases in VAF post-treatment aligned with clinical stabilization, whereas rising or persistent VAFs reflected disease progression, therapeutic resistance, or evolving clonal mutations. Elevated VAFs correlated strongly with clinically confirmed LMD and were concordant with radiographic and clinical indicators of disease. **Conclusions**: VAF analysis in CSF provides a quantitative biomarker for the detection and monitoring of metastatic CNS disease. These findings support its utility as a complementary tool to conventional diagnostics, offering real-time insights into disease burden, therapeutic response, and clonal evolution in LMD.

## 1. Introduction

Central nervous system (CNS) metastases represent a significant challenge in advanced cancer management, occurring in approximately 20–40% across various solid tumors. This involvement includes parenchymal brain metastases, spinal cord lesions, and leptomeningeal disease (LMD), each carrying distinct diagnostic and therapeutic implications [1,2]. Brain metastases arise when tumor cells colonize the brain parenchyma, while leptomeningeal metastases are characterized by the dissemination of tumor cells within the cerebrospinal fluid (CSF) and along the leptomeninges, the delicate membranes surrounding the brain and spinal cord [1,3]. CNS involvement may also be presented as epidural or dural metastases, although these are less common [4]. The development of CNS metastases significantly impacts morbidity and mortality, given the brain’s limited capacity for compensatory function and the unique challenges posed by the blood–brain barrier in delivering systemic therapies [1,4].

Among CNS complications, LMD represents a distinct and particularly aggressive form of disease. Though relatively rare, LMD occurs in approximately 5–8% of patients with metastatic solid tumors, most commonly in breast cancer, non-small cell lung cancer (NSCLC), and melanoma [5]. Beyond differences in clinical presentation, genomic profiling confirms that LMD displays molecular divergence from matched parenchymal brain metastases, reflecting microenvironmental adaptation and therapeutic selection pressures [3]. In addition to metastatic solid tumors, LMD also arises in select CNS tumors, such as primary CNS lymphoma, medulloblastoma, and ependymoma, particularly in pediatric and young adult populations [6]. Advances in systemic cancer therapies have extended survival across multiple tumor types. However, improved systemic disease control has led to an increased incidence of CNS complications, including LMD, as the CNS often functions as a sanctuary site due to the limited penetration of many therapeutic agents across the blood–brain barrier. As a result, LMD is increasingly encountered as a site of disease relapse, often in later stages when treatment options are limited [7].

LMD is often underdiagnosed and remains a significant clinical challenge. Patients frequently present with nonspecific neurological symptoms, including headache, cognitive changes, cranial neuropathies, or radiculopathies, which can overlap with other CNS complications of advanced cancer and further complicate timely recognition [8]. Magnetic resonance imaging (MRI) with contrast may reveal leptomeningeal enhancement, yet 20–30% of LMD cases can have normal MRI scans or inconclusive findings, particularly in early disease or low tumor burden [5]. In addition, radiographic interpretation can be limited by subtle or patchy enhancement patterns and interobserver variability, reducing diagnostic confidence in borderline cases [8]. Although CSF cytology is traditionally considered the diagnostic gold standard, it only has a sensitivity of about 70% when used for initial diagnosis and often requires repeated lumbar punctures for diagnosis, resulting in a delay in diagnosis [9]. Even with serial sampling, false-negative results are common, especially in patients with focal or low-volume leptomeningeal involvement [8]. Cytology also does not provide molecular information about the cancer, even when the result is positive. As a result, cytologic analysis cannot inform tumor biology, therapeutic targeting, or mechanisms of treatment resistance. These limitations can result in delayed treatment and suboptimal outcomes, emphasizing the need for more sensitive, molecular-based, less invasive diagnostic tools.

Given these limitations, there is growing interest in molecular-based, minimally invasive diagnostic tools for LMD. Liquid biopsy approaches have gained increasing attention for their ability to detect and monitor malignancy through tumor-derived material in biofluids [10]. These approaches enable minimally invasive assessment of tumor burden, molecular evolution, and treatment response over time, overcoming many of the limitations associated with tissue-based biopsies [11]. In the context of CNS involvement, CSF-derived circulating tumor DNA (ctDNA) offers a promising avenue due to its direct access to the leptomeningeal space and ability to provide high-resolution molecular insights [12]. Unlike plasma ctDNA, which is limited by the blood–brain barrier, CSF ctDNA has demonstrated superior sensitivity in detecting CNS disease [12]. Among the new technologies evolving in this space, the Belay [13] CSF liquid biopsy assay, which utilizes MethylSaferSeq [14], enables the simultaneous detection of genomic (gene-level alterations-Summit™ [13] and chromosomal arm-level alterations-Ascent™) and epigenomic (*MGMT* promoter methylation-Vantage™) [15] alterations in tumor-derived DNA (tDNA) from CSF of primary and metastatic CNS cancers, with high sensitivity and specificity in a single workflow.

One of the most informative metrics in ctDNA analysis is the variant allele frequency (VAF) of gene alterations, which quantifies the proportion of DNA sequencing reads containing a specific tumor-associated mutation [16]. VAF is influenced by tumor burden, clonal architecture, and the extent of tumor DNA release into the biofluid [17]. VAF has been shown to correlate with tumor burden, therapeutic response and the emergence of therapeutic resistance in systemic malignancies, and serial monitoring of plasma ctDNA VAF is increasingly used to assess therapeutic efficacy and detect minimal residual disease [18,19]. The VAF threshold for calling a variant in ctDNA depends on the assay and sequencing depth. Many platforms show high sensitivity and reproducibility for variants with VAF ≥ 0.5%, whereas detection becomes less reliable below this level [20,21]. Some highly optimized assays can detect variants down to ~0.25% VAF, although sensitivity declines substantially below that point [22]. Nonetheless, its clinical utility in monitoring disease progression or informing the diagnosis of LMD has not been fully established in the context of liquid biopsy assays. The hypothesis underlying its potential application is that increased tumor shedding into the CSF would correspond with higher VAFs, particularly in disease progression, development of resistance to therapy and aggressive or widespread leptomeningeal involvement [23]. However, VAF interpretation must account for several pre-analytical variables, including CSF collection timing relative to treatment cycles, sample volume, cell-free DNA concentration, and processing delays, all of which can impact the detection and quantification of tumor-derived DNA.

This study sought to evaluate the role of CSF ctDNA VAF, measured using the Summit™ assay, as a diagnostic and longitudinal biomarker for LMD, contributing to the growing and exciting body of literature in this field.

## 2. Methods

### 2.1. Study Cohort and Specimen Collection

A total of 118 CSF specimens were analyzed, comprising 112 unique patient samples and six repeat specimens. Samples were submitted for Summit™ 1.0 testing from patients with a provisional diagnosis of metastatic CNS cancer. The cohort included patients with a range of primary malignancies, including breast cancer, lung cancer, and other solid tumors, reflecting the clinical diversity of cancers associated with CNS involvement.

Clinical information provided at the time of testing was used to categorize specimens into three groups: confirmed LMD, suspected LMD, or parenchymal CNS metastases without evidence of leptomeningeal involvement. For a subset of patients, serial CSF samples were available, enabling longitudinal analysis.

### 2.2. Longitudinal Analysis

Repeat specimens received for tracking therapy response were analyzed in 14 cases (total of 30 specimens) to assess VAF dynamics in relation to therapeutic response and disease progression. The study was approved by the Advarra Institutional Review Board (Pro00088250) and was performed in accordance with the Declaration of Helsinki and the Health Insurance Portability and Accountability Act (HIPAA).

### 2.3. CSF Liquid Biopsy and Molecular Analysis

VAF of gene-level variants detected by the Summit™ CSF liquid biopsy assay in a cohort of 118 Summit™ positive specimens was evaluated in the context of clinical presentation, neuroimaging, and CSF cytology to inform diagnosis of LMD. The Summit™ assay detects gene-level somatic alterations, including single-nucleotide variants (SNVs), multi-nucleotide variants (MNVs), and insertions and deletions (indels), across a targeted panel of 32 cancer-related genes. The panel does not include the most common CHIP-associated genes, such as *DNMT3A*, *TET2*, or *ASXL1*, reducing the contribution of CHIP-derived variants to VAF measurements. VAF was calculated as the proportion of sequencing reads supporting the variant allele relative to the total reads covering the corresponding genomic locus using the Summit-targeted next-generation sequencing assay. Based on analytical validation of the Summit platform, variants were reported at a VAF threshold of ≥0.3% [13], and detection does not correlate with DNA input or CSF volume.

## 3. Results

### 3.1. Molecular Findings Across the Cohort

**Figure 1:**
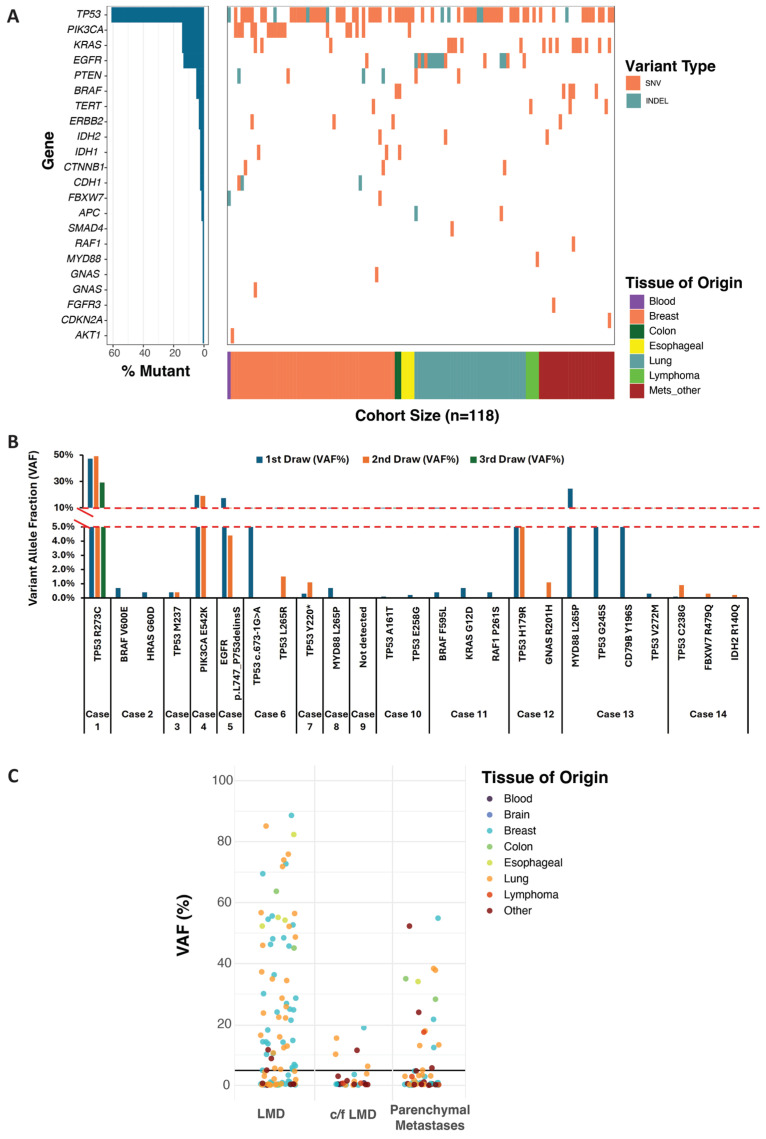
Clinically significant variants were detected in 22 of the 32 genes included in the Summit panel, with frequencies varying by tissue of origin. SNV, single nucleotide variant; INDEL, insertion/deletion; CNS, central nervous system (**A**). VAF of clinically significant variants detected in 14 cases (30 specimens collected over time) was used to monitor disease progression and therapeutic response (**B**). Distribution of variant allele frequency (VAF) across clinical groups including leptomeningeal disease (LMD), concern for LMD (c/f LMD), and parenchymal metastases. The horizontal black line indicates the 5% VAF threshold, which was used as a cutoff associated with the diagnosis of LMD. VAF may serve as a quantitative measure to inform the diagnosis of LMD and to monitor disease progression or response to therapy (**C**).

To demonstrate the clinical utility of VAF to track therapy response, 118 CSF specimens (112 unique samples and 6 repeat specimens) that were received for Summit™ 1.0 testing from patients with a provisional diagnosis of metastatic CNS cancer were evaluated. All 118 specimens had clinically significant gene-level variants (SNVs, MNVs and Indels) identified in 22 of the 32 genes on the panel (Figure 1A) and included 50 breast, 35 lung and 33 other primary tissue cancers. Across the cohort, the most frequently detected alteration was *TP53* (*n* = 87), followed by *EGFR* (*n* = 19), *KRAS* (*n* = 17), and *PIK3CA* (*n* = 17). Other recurrent alterations included BRAF (*n* = 6), *PTEN* (*n* = 6), and *TERT* (*n* = 5), while *ERBB2* (*n* = 4) and *CDH1*, *CTNNB1*, *IDH1*, and *IDH2* (*n* = 3 each) were observed less frequently. Mutations in *APC*, *FBXW7*, and *GNAS* were detected in two samples each, whereas *AKT1*, *CDKN2A*, *FGFR3*, *MYD88*, *RAF1,* and *SMAD4* were each detected in a single sample (Figure 1A).

### 3.2. Longitudinal VAF Dynamics and Therapy Response

Monitoring CNS cancer progression or therapy response requires serial evaluation of CSF over time. Serial CSF samples from 14 patients, comprising 30 specimens collected over time, were evaluated for VAF dynamics (Figure 1B). Of the 14 patients, only two underwent three repeated tests, while the remaining patients had two tests each. The intervals between sample collections varied widely, ranging from 13 to 111 days (Supplementary Table S1). Analysis of VAF dynamics revealed distinct patterns associated with clinical outcomes. In seven cases, follow-up samples demonstrated a decreased VAF or complete absence of detectable variants following therapy. These molecular changes correlated with clinical improvement and ongoing treatment response, supporting the use of VAF as a real-time indicator of therapeutic efficacy [18]. The specimens demonstrated stable VAFs over time, which could represent either stable disease or therapeutic resistance. In one case (#7), an increase in VAF likely corresponded with disease progression. Three cases revealed the emergence of a new somatic variant on repeat sampling, suggesting clonal evolution or spatial heterogeneity within the leptomeningeal compartment [24]. These findings highlight the dynamic nature of CNS disease and the ability of CSF ctDNA analysis to capture evolving tumor genomics. One case (#9) had no detectable variants across serial samples, reflective of either low disease burden or limitations in ctDNA release into the CSF (Figure 1B).

These longitudinal observations highlight the potential utility of VAF as a real-time tool for assessing treatment response and tracking disease evolution. Rising VAFs may serve as early indicators of progression, potentially preceding clinical or radiographic evidence by weeks or months. Conversely, decreasing or stable VAFs following therapy may reflect effective disease control and therapeutic response. This dynamic behavior mirrors trends seen in other malignancies where serial ctDNA measurements have been used to evaluate clonal evolution, therapeutic response, and resistance emergence [18,24].

### 3.3. VAF as a Diagnostic Tool for LMD

To further demonstrate the potential of VAF as a quantitative measure to inform the diagnosis of LMD, all 118 specimens were clinically categorized based on clinical information provided at the time of testing as confirmed or suspected LMD or parenchymal metastases without leptomeningeal spread. Stratification by clinical context revealed a distinct pattern, with 42 of 61 (69%) samples with confirmed LMD exhibiting ≥ 5% VAFs (Figure 1C). Lower VAFs (<5%) were generally observed in patients with suspected LMD (77%, 13 of 17 specimens) or parenchymal CNS disease without clear leptomeningeal involvement in 62.5% (25 of 40 specimens) (Figure 1C). However, among patients with suspected but unconfirmed LMD (24%), elevated VAFs often served as early molecular evidence of disease.

In several cases where initial imaging and CSF cytology were inconclusive, the detection of high VAF prompted further clinical investigation, which ultimately led to a confirmed diagnosis of LMD. Similarly, among the patients initially classified as having parenchymal metastases only or with concern for (c/f) or suspicion of LMD, ten cases with VAFs > 5% were later reclassified as LMD based on subsequent clinical or pathological findings (Table 1A). These observations suggest that a VAF threshold of ≥5% may represent a clinically meaningful marker for diagnosing LMD, particularly in cases where traditional diagnostic modalities fall short. This proposed threshold aligns with the biological rationale that increased tumor DNA shedding into the CSF reflects active leptomeningeal involvement and is consistent with similar quantitative benchmarks established in plasma ctDNA studies [23], although such thresholds have not yet been widely validated for use in CSF. Supporting this interpretation, unpublished data from a prior cohort demonstrated that VAFs ≥ 5% in CSF samples from patients with c/f or suspicion of LMD were clinically confirmed by the treating physician as LMD. Furthermore, in a small subset of cases in which LMD was not confirmed, molecular profiling revealed concordant variants between primary tumors and CSF samples (Table 1B). This concordance supports a shared clonal origin and is consistent with prior reports indicating that CSF-derived ctDNA accurately captures the genomic landscape of leptomeningeal disease [25]. Collectively, these findings align with existing literature suggesting that CSF ctDNA VAF may serve as a complementary biomarker for LMD detection and disease assessment, while underscoring the need for standardized thresholds and prospective clinical validation.

## 4. Discussion

This study demonstrates that variant allele frequency derived from CSF liquid biopsy is a clinically meaningful biomarker for both the diagnosis and monitoring of leptomeningeal disease. The data support two primary applications of VAF: longitudinal assessment of therapeutic response and quantitative support for LMD diagnosis, particularly in cases where conventional diagnostic modalities are inconclusive.

The results reinforce the validity of using VAF as a quantitative measure to distinguish LMD from parenchymal metastatic disease in CSF-derived ctDNA. It offers a more sensitive and objective approach in diagnosing LMD in clinical scenarios where MRI findings are equivocal or CSF cytology is negative, both of which are known limitations in early or low-burden disease. Furthermore, a VAF threshold of ≥5% emerged as a potentially useful marker, aligning with confirmed LMD and supporting clinical evaluation in cases of ambiguity. Notably, a VAF threshold of ≥5% emerged as a potentially useful marker for identifying confirmed LMD, offering additional support in clinically ambiguous cases. Although preliminary, this threshold is biologically plausible, reflecting the expectation that higher levels of tumor DNA in CSF correspond to more extensive leptomeningeal involvement [26]. However, the establishment of such quantitative cutoffs could standardize interpretation across centers, reducing diagnostic variability and enabling more timely initiation of targeted therapies.

Additionally, the study highlighted the value of longitudinal VAF measurement as a dynamic biomarker to track CNS disease status and therapeutic response over time. This approach mirrors the increasing use of serial plasma ctDNA monitoring in systemic cancers to guide treatment decisions and assess efficacy. The ability to repeatedly sample CSF and quantitatively track VAF changes may allow for earlier detection of treatment resistance or disease progression, potentially improving patient outcomes. Integrating VAF monitoring with clinical and radiographic assessment could enhance risk stratification and support more precise, real-time therapeutic decision-making, reflecting the broader trend of using serial ctDNA measurements to inform systemic cancer management [16].

Taken together, these findings provide a foundation for integrating VAF analysis into CSF liquid biopsy workflows. A quantitative threshold of ≥5% may help inform diagnostic decisions, while longitudinal monitoring could guide treatment evaluation. While further validation in larger, prospective tumor-specific cohorts is needed, this work provides a foundation for integrating VAF into CSF liquid biopsy testing workflows. As CSF-based technologies such as the Summit™ assay continue to evolve, they hold promise for improving the precision and timeliness of care in patients with CNS malignancies.

## 5. Conclusions

CSF-derived VAF is a clinically meaningful biomarker for both diagnosing and monitoring leptomeningeal disease. A threshold of ≥5% provides quantitative support for diagnosis, while longitudinal tracking of VAF offers dynamic insights into therapeutic response and disease progression. Integrating VAF analysis into CSF liquid biopsy workflows holds promise for more sensitive, timely, and personalized management of CNS malignancies. However, these findings are preliminary and require validation in larger, prospective, tumor-specific cohorts before widespread clinical implementation.

## Figures and Tables

**Table 1:** Demonstrating the validity of VAF as a quantitative measure in CSF.

A. Clinical Follow-Up of Summit High-VAF Cases for LMD Determination			
**Study ID**	**Tissue of Origin**	**LMD Status**	**Clinical Follow-Up**
7	Breast	c/f LMD	Confirmed LMD
47	Lung	c/f LMD	Confirmed LMD
53	Breast	Parenchymal Mets	Confirmed LMD
146	Esophageal	Parenchymal Mets	Confirmed LMD
147	Lung	c/f LMD	Confirmed LMD
172	Pancreas	c/f LMD	Confirmed LMD
178	Lung	c/f LMD	Confirmed LMD
216	Esophageal	Parenchymal Mets	Confirmed LMD
312	Gastric	c/f LMD	Confirmed LMD
388	Breast	c/f LMD	Confirmed LMD
B. Summit Variant Concordance with Prior Tumor Biopsy Results (Variants limited to 32 genes evaluated by Summit™			
**Study ID**	**Tissue of Origin**	**Gene-Level Variants Detected by Summit**	**Variants Detected in Prior Primary or Metastatic Tumor**
94	Lung	EGFR L858R	EGFR L858R
114	Lung	EGFR L746_S752delinsV TP53 R213fs,	EGFR L746_S752delinsV TP53 R213fs,
307	Lung	EGFR L858R	EGFR L858R

c/f—concern for; LMD—leptomeningeal disease.

## Data Availability

The original contributions presented in this study are included in the article. Further inquiries can be directed to the corresponding author.

## Supplementary Materials

The following supporting information can be downloaded at: https://www.mdpi.com/article/10.3390/diagnostics16060851/s1, Supplementary Table S1: Sampling intervals and VAF of variants detected (n = 14).
