# Supplementary material for: Variant Allelic Frequency to Track Therapy Response and Evaluate Leptomeningeal Disease in Metastatic Central Nervous System Cancers
Source: Diagnostics (Basel). 2026 Mar 13;16(6):851. doi: 10.3390/diagnostics16060851 (PMC13025417; doi:10.3390/diagnostics16060851)
Supplement: Supplementary file 1 [file diagnostics-16-00851-s001.zip › diagnostics-4127662-supplementary.pdf]

Supplementary Table S1

Patient ID	Number of CSF Samples	Sampling Interval (days)	Detected Mutation/Gene	VAF Sample 1 (%)	VAF Sample 2 (%)	VAF Sample 3 (%)
Case 1	3	42, 77	TP53 R273C	52.30%	54.20%	34.10%
Case 2	2	96	BRAF V600E	0.70%	0	
			HRAS G60D	0.40%	0	
Case 3	2	54	TP53 M237	0.40%	0.40%	
Case 4	2	77	PIK3CA E542K	24.80%	24.10%	
Case 5	2	70	EGFR p.L747_P753delinsS	22.40%	4.40%	
Case 6	2	28	TP53 c.673-1G>A	5%	0.00%	
			TP53 L265R	0.00%	1.10%	
Case 7	2	14	TP53 Y220*	0.30%	1.10%	
Case 8	2	107	MYD88 L265P	0.70%	0.00%	
Case 9	2	111	Not detected	0.00%	0.00%	
Case 10	3	78, 96	TP53 A161T	0.10%	0.00%	0.00%
			TP53 E258G	0.20%	0.00%	0.00%
Case 11	2	87	BRAF F595L	0.40%	0.00%	
			KRAS G12D	0.70%	0.00%	
			RAF1 P261S	0.40%	0.00%	
Case 12	2	92	TP53 H179R	13.70%	5%	
			GNAS R201H	0%	1.10%	
Case 13	2	84	MYD88 L265P	29.60%	0%	
			TP53 G245S	15%	0%	
			CD79B Y196S	14.10%	0%	
			TP53 V272M	0.30%	0%	
Case 14	2	13	TP53 C238G	0.10%	0.90%	
			FBXW7 R479Q	0%	0.30%	
			IDH2 R140Q	0%	0.20%	
